# Screening of cervical cancer in Catalonia 2006–2012

**DOI:** 10.3332/ecancer.2015.532

**Published:** 2015-04-29

**Authors:** Silvia de Sanjosé, Raquel Ibáñez, Vanesa Rodríguez-Salés, Mercè Peris, Esther Roura, Mireia Diaz, Aureli Torné, Dolors Costa, Yolanda Canet, Gemma Falguera, Maria Alejo, Josep Alfons Espinàs, F. Xavier Bosch

**Affiliations:** 1 Cancer Epidemiology Research Programme, Catalan Institute of Oncology–IDIBELL, L’Hospitalet de Llobregat, 08907, Spain; 2 CIBER en Epidemiología y Salud Pública (CIBERESP), Barcelona 08036, Spain; 3 Instituto Clínico de Ginecología, Obstetricia y Neonatología, Hospital Clínic, Institut d’Investigacions Biomèdiques August Pi i Sunyer, Facultad de Medicina, Universidad de Barcelona, Barcelona 08036, Spain; 4 Coordinación Maternoinfantil y Atención a la Salud Sexual y Reproductiva, Institut Català de la Salut, Bacelona 08007, Spain; 5 Corporació Sanitària Parc Taulí, Sabadell 08208, Spain; 6 Atención a la Salud Sexual y Reproductiva (ASSIR) Vallès Oriental y Occidental, Dirección de Atención Primaria, Gerencia Territorial Metropolitana Norte, Institut Català de la Salut, Sabadell, Barcelona 08202, Spain; 7 Consorci Sanitari Integral, Hospital General de l’Hospitalet, L’Hospitalet de Llobregat, Barcelona 08906, Spain; 8 Unidad de Registro de Cáncer de Cataluña, Departamento de Salud, Autoridad Regional de Cataluña, L’Hospitalet de Llobregat, Barcelona 08907, Spain

**Keywords:** cervical cancer, screening, coverage, HPV

## Abstract

The early detection of intraepithelial lesions of the cervix, through the periodic examination of cervical cells, has been fundamental for the prevention of invasive cervical cancer and its related mortality. In this report, we summarise the cervical cancer screening activities carried out in Catalonia, Spain, within the National Health System during 2008–2011. The study population covers over two million women resident in the area. The evaluation includes 758,690 cervical cytologies performed on a total of 595,868 women. The three-year coverage of cervical cytology among women aged between 25 and 65 years was 40.8%. About 50% of first screened women with negative results had not returned to the second screening round. The introduction of high-risk human papillomavirus DNA (HPV) detection, as a primary screening cotest with cytology among women over age 40 with a poor screening history, significantly improved the detection of cervical intraepithelial neoplasia grade 2 or worse (CIN2+), being far superior to cytology alone. Cotesting did not improve the detection of CIN2+. The use of the HPV test for the triage of atypical squamous cell undetermined significance (ASC-US) improved the selection of women at high risk of CIN2+.

Sampling (both cytology and HPV test) was largely performed by midwives (66.7%), followed by obstetricians (23.8%) and nurses (7%). Over half of the centres (54.8%) had full use of online medical records. During the study period, educational activities for professionals and for women were carried out periodically.

The organisation of screening as a population activity in which women are actively called to the screening visit and the introduction of HPV testing as a primary screening tool are strongly recommended to ensure the maximum population impact in the reduction of the cervical cancer burden.

## Introduction

The early detection of intraepithelial lesions of the cervix through the periodic examination of cervical cells has been fundamental for the prevention of invasive cervical cancer and its related mortality [1]. The impact of cervical cancer screening programmes in the target population has resulted in an important decline of the disease burden [2]. However, many European countries have non-organised approaches to cervical cancer prevention measures, and screening is performed on an opportunistic basis [3]. In Spain, cervical cancer screening is opportunistic with an estimated coverage of 70% when both private and public sector are considered [4]. However, there is an irregular approach to screening intervals, age of recommendation, and no systematic evaluation of coverage [5]. Recently, seven Spanish scientific societies have jointly recommended the implementation of cervical cancer screening under an organised structure. This could allow women to be followed for adequate call and recall to screening and would maximise the available use of resources [6].

In 2006, the autonomic region of Catalonia established specific guidelines for the prevention of cervical cancer within the public health sector [7], with a clear aim to regulate the interval between cervical cytologies, to increase cervical cancer coverage among underscreened women and to introduce human papillomavirus DNA (HPV) testing in selected population categories.

The aim of this report was to provide a summary evaluation of the activities performed during the period 2006–2012 to facilitate better planning for new updated guidelines. For this, we estimated the coverage of screening, the interval between cytologies within a first screening round and the rescue of underscreened women. We also evaluated the value of HPV testing as a cotest with cervical cytology in the underscreened women and in the follow-up of women with a diagnosis of atypical squamous cell of undetermined significance (ASC-US) [8–12]. Finally, organisational aspects of the care for sexual and reproductive health centres (ASSIRs) were evaluated.

## Material and methods

The information was derived from the following sources:

**1. Information system of primary care services (SISAP)**

The SISAP reports the medical care activities within the primary health care system of 75% of the residents in Catalonia. Through this system, we were able to evaluate the coverage, the interval between cytologies, the number of HPV tests requested and the results of the different tests by age categories for centres requesting the exams, health regions, and for the overall population of Catalonia. We included information on women over age 15 years. The evaluation of the interval between cytologies was measured as the follow-up of women that were screened for the first time in 2008 and had a normal cytology test as specified elsewhere [8]. All the information provided was anonymised. Additionally, for the years 2006–2009, a specific registration of the number of HPV tests performed was available to monitor its new introduction. This specific registration was stopped once the medical records and the manual form proved to be highly concordant.

**2. Pathology departments**

Women with the criteria of underscreened or with ASC-US were followed up for HPV results. Six pathology departments provided data on the tests (HPV test, cytologies, and biopsies) for at least three years after the index visit. Laboratories provided data on the follow-up of selected women meeting the criteria of (i) being above 39 years old with no previous cytology in the previous 5 years (underscreened women) or (ii) having a ASC-US diagnosis within a period of 3 months. The laboratories included were Hospital Universitario Dr. Josep Trueta, Consorcio hospitalario de Vic, Hospital Universitario Joan XXIII, Hospital del Mar, Hospital universitario de Bellvitge, Hospital de Granollers, Hospital de Althaia, and Laboratorio de Atención Primaria Dr. Robert. The main outcome was histological confirmation of cervical intraepithelial neoplasia grade 2 or worse (CIN2+) [10, 11]. The risk of CIN2+ was evaluated following a Kaplan–Meier approaches.

**3. Interlaboratory reproducibility of performance of hybrid capture 2 (HC2) on cervical specimens**

During the period 2008–2011, all the laboratories for HPV screening in Catalonia (Hospital Universitario Dr. Josep Trueta, Consorcio Hospitalario de Vic, Hospital Universitario Joan XXIII, Hospital del Mar, Hospital Universitario Bellvitge-Institut Català d’Oncologia, Hospital Clínic, Hospital Universitario Vall d’Hebron, Hospital Universitario Verge de la Cinta, Hospital Universitario Arnau de Vilanova, Consorcio Sanitario Parc Tauli y los laboratorios de Atención Primaria Doctor Robert y Bon Pastor) were requested to provide samples for the proficiency testing survey in order to monitor the interlaboratory reproducibility of performance of HC2 assay (Qiagen, Gaithersburg, MD). Every year, 20 samples stratified by different value categories were retested in a different laboratory from the original one. Concordance analyses were performed through the Kappa statistics and by linear regression as detailed elsewhere [12].

**4. Organisation of the ASSIRs**

A questionnaire was sent to all ASSIRs to evaluate the use of the 2006 recommendations; the composition of the screening teams, the activities done to identify underscreened women, and the availability of adequate resources to gather the information. Overall, 52.4% of professionals who answered the survey were obstetricians and 47.6% were midwives.

The evaluation was approved by the IRB of the Instituto de Investigación Biomédica de Bellvitge.

Cytological results were classified using the Bethesda system [13].

## Results

### Cytology: coverage, interval, and immigration

**Figure 1.**
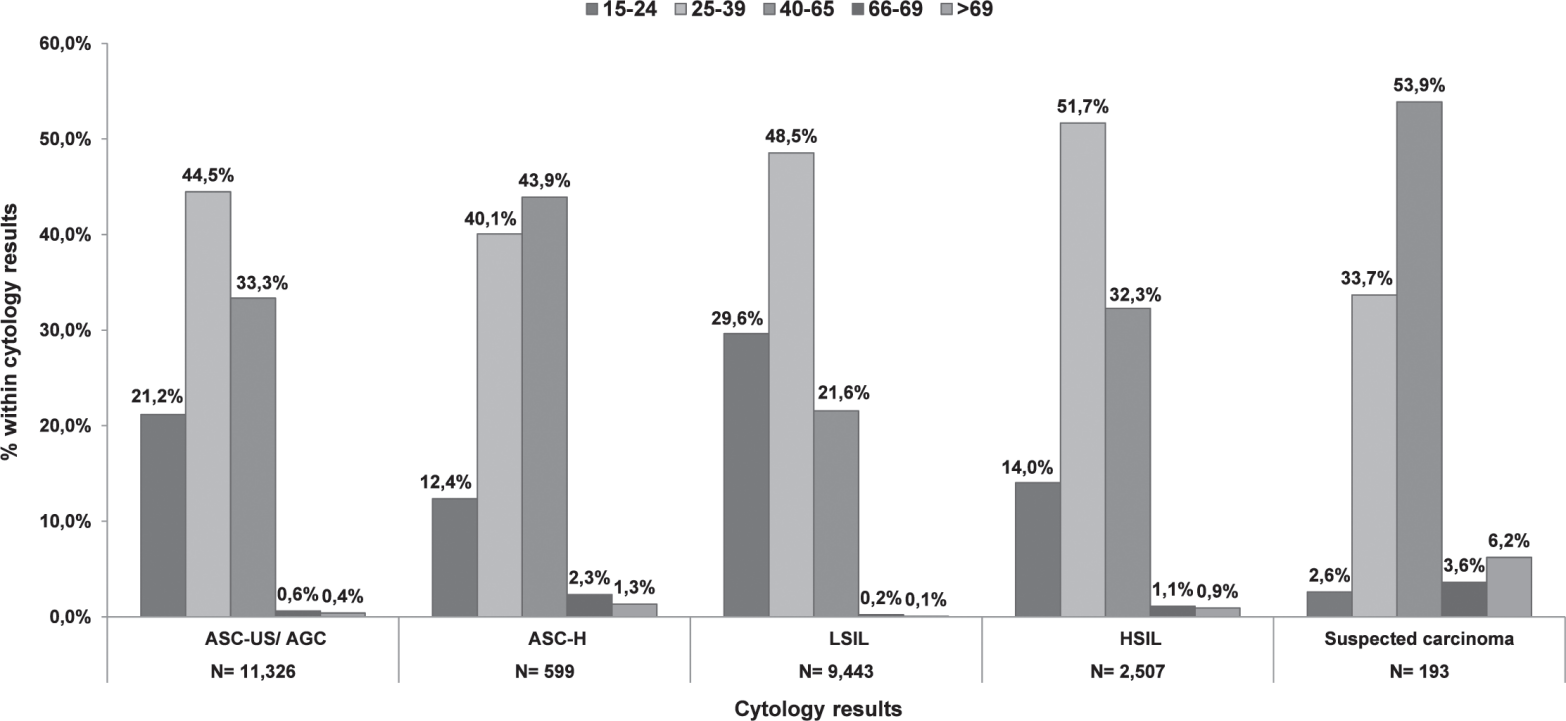
Distribution of abnormal cytology for the period 2008–11 by age group in Catalonia.

In Catalonia, during the period 2008–2011, within the National Health System (NHS), 758,690 smears were performed on a total of 595,868 women over 15 years old. Every cytology within a 3-year interval was registered in 40.8% of the women aged between 25 and 65 years visiting the health care facilities at least once (Table 1). There was certain variability in the coverage by health regions, the Barcelona health area (Barcelona City, Barcelona South and North) being the one with a higher coverage. Taking one cytological result per woman (the most advanced one in case of having more than one), during an interval of three years, 3.7% of cervical smears were positive for cervical intraepithelial lesions. Of these, 44.8% were ASC-US/atypical glandular cell (AGC), 2.3% for atypical squamous cells that cannot exclude a high-grade lesion (ASC-H), 42.9% for squamous intraepithelial lesion low grade (LSIL), 9.4% squamous intraepithelial high-grade lesion (HSIL), and 0.7% to suspected carcinoma. Figure 1 shows the distribution of cervical lesions by age group. The large volume of ASC-US/ AGC, LSIL, and HSIL was located in the age group of 25–39 years, while suspected carcinoma was more common in women who were 40–65 years. Among women having a first negative test, the average interval to the second following test was estimated to be around 2.4 years. Approximately 50% of women with a negative cytology did not return for a second round of screening in the 3-year period [8].

Women born outside Spain were considered immigrant population. Among the 91,427 immigrant women, 115,488 cervical cytologies were performed. By place of birth, 51% of women were from Morocco, Ecuador, Bolivia, Colombia, and Romania. It was observed that the cytological coverage and the proportion of positive cytologies among the population of 25–65 years of age were statistically significantly higher among immigrants than Spanish women (51% vs. 39% and 4.5% vs. 3%, respectively). Women from the regions of North America (6.9%), Central America (6.8%), and Western Europe (5.8%) had a higher prevalence of abnormal cytologies, while women from Oceania (0%) and North Africa (2.6%) had the lowest percentages of abnormal cytologies. On the other hand, participation in the second round of screening among women with normal cytology was lower among immigrant women (43.1%) compared with Spanish women (50.7%) [9].

## The evaluation of the HPV test

During the period 2006–2012, a total of 116,970 HPV tests were performed in Catalonia. Most of the HPV tests were performed in women identified as being underscreened (68.5%) of which 6.3% were HPV positive. The second most common request was for the triage of ASC-US (24%) with a percentage of positivity of 48.1%.

The evaluation of cotesting was performed on a group of 1,832 underscreened women with a mean age of 54.1 years (range 40–88 years) [10]. The majority (92.4%) had both negative screening tests and 338 women were recommended to stop screening for being over 65 years old with a negative cotesting. Thus, we followed 1,494 women for at least 3.5 years, of which only 767 women (51.3%) returned to the second screening round. The greatest loss of follow-up was among women with both tests negative, compared with those who had at least one positive test (p < 0.05).

At baseline, 2.2% of women had a positive cytology result, 6.7% a positive HPV test, and 1.3% had both tests positive. At the end of the follow-up period, there were nine cases of histologically confirmed CIN2, seven CIN3, and two cancers (18/767, 2.3%). All but one of the CIN2 cases was HPV positive. The two cancer cases (a squamous cell carcinoma in stage II and an adenocarcinoma in stage I) had associated a baseline cytology of ASC-US and AGC, respectively; however, 56% of cases of CIN2/3 had normal cytology at baseline. The evaluation of both tests resulted in a very poor sensitivity of cytology as compared to HPV tests for CIN2+ (44.4 vs. 94.4). However, the specificity and PPV were higher in cytology (96.4 and 22.9, respectively) compared to the values of the HPV alone or the combine results of cotesting. The negative predictive value (NPV) was high in any of the three strategies (Table 2) [10].

We evaluated the triage of ASC-US using HPV test for the prediction of CIN2+ in 611 women with a mean age of 34.5 years (range 15–79 years) [11]. Among these women, we obtained an adequate follow-up for 493 (80.7%) women, of which 48.3% were positive for HPV. Almost all lesions histologically confirmed of CIN2+ were identified in the HPV-positive group (35/36). In this group, two squamous cell carcinomas (stage II) and a mucinous adenocarcinoma (stage III) were identified. None of the women with a negative HPV had a diagnosis of CIN3. Only one case (0.4%) was diagnosed with CIN2. The sensitivity of the HPV test to detect CIN2+ was 97.2% (95% CI: 85.5–99.9) with a specificity of 68.3% (CI: 63.1–73.2) [11].

Finally, for the purpose of monitoring the interlaboratory reproducibility of performance of HPV detection technique, a total of 946 samples were re-tested in a second laboratory during the period 2008–2011. A total of 44 (4.6%) discrepancies were found, and the overall correlation coefficient between the two measurements was 0.95 (p < 0.05) for the continuous evaluation of RLU values, while the kappa value for positive/negative was 0.91, demonstrating an almost excellent interlaboratory agreement for all 12 participating laboratories [12].

## Organisational structure in ASSIR

Among all ASSIR centres, 83.3% declared to follow the 2006 protocol. ASSIR centres reported that cervical cancer screening activities involved multidisciplinary professionals. In 52% of the ASSIR centres, midwives were responsible for the identification of women susceptible to cervical cancer screening. In 23.8% of the centres, the administrative staff helped in detecting underscreened women through the routine registration of cytologies. Sampling (both cytology and HPV test) was largely performed by the midwives (66.7%), followed by obstetricians (23.8%) and nurses (7%). The communication of Pap results was carried out by obstetricians and midwives in similar percentages (50%, 40.5%, respectively), while communicating the results of HPV tests was performed more often by obstetricians (59.5%) compared to midwives (35.7%).

About 5% of the centres reported warning systems to identify women who were not routinely screened. Some centres referred that coordination with primary health care teams facilitated the recruitment of women with inadequate screening, while highlighting the facilitating role of social entities working with socially disadvantaged groups. Almost all centres (97.6%) reported specific activities (ex. telephone call) to contact women with an abnormal cytology or a positive HPV test.

Most centres (54.8%) used the online medical records that were compatible with the systems of other centres, allowing an automatic download. However, still less than 40% of the centres had this technology available. Specifically, the results from 40.5% of cytologies, 28.6% of HPV testing, 40.5% of colposcopy, and 35.7% of biopsies were registered manually. In 7% of the centres, cytological results were communicated via email.

In most centres (97.6%), indicators related to screening for cervical cancer were part of the NHS yearly quality evaluation of the centres. Also, 73.8% of centres trained their professionals on preventive activities including cervical cancer prevention, although educational programmes were reported on an irregular basis.

## Information and training of health professionals

During the period 2006–2012, there were two information campaigns aimed at the general population, and many scientific conferences were held for professionals at the primary health level at the start of the protocol implementation. A free online course was offered to professionals involved in screening, in order to reinforce the contents of the protocol and its applicability in clinical practice. The course also addresses basic knowledge about the HPV vaccine. A total of more than 1,500 professionals in Catalonia were enrolled. Further, the detailed protocol was available on the website of Canal Cancer of the Health Department (http://www20.gencat.cat/portal/site/cancer) [14].

Leaflets were distributed to all centres to increase awareness in the general population in the Catalan and Spanish languages. Furthermore, dissemination activities in the regular press and in the local radio channel were occasionally conducted.

## Discussion

This report summarises the cervical screening coverage within the NHS in Catalonia, Spain. Data are based on electronic medical records of over two million women including cervical cytologies and/or HPV tests perfomed per women and in some instances including also pathology records [15]. The selected use of the HPV test as primary screening or as a triage test resulted in a diagnosis of an increased volume of cervical pathology CIN2+ as compared to the use of cervical cytology. The HPV test improved the management of women with an ASC-US diagnosis as it selected those women with a risk of CIN2+ at 3 years among those that tested positive for HPV.

After evaluation of over 750,000 cytologies performed in the study period, the coverage per women in a three-year period was estimated to be 40% among those aged 25–65 years. In an ancillary study, when non-attenders were asked about the reasons for not attending the NHS centres, 30% of women reported visiting a private gynaecologist and largely on an annual basis [5, 16]. Thus, these data indicate that the real cervical cancer screening coverage in Catalonia is likely to be around 70% and that a group of women may have been screened more often than recommended. There remain approximately 30% of women with no screening at all. Recent data [16] confirmed that organised screening could not only help to increase coverage but also would greatly reduce the number of screening tests in line with European and Spanish recommendations from scientific societies [6, 17–19]. The organisation of screening could also reduce social differences in our immigrant population, where the regularity of screening was lower than that observed in the Spanish population [9].

Through the follow-up of screened women, we were able to identify that about half of the women that were screen-negative at first round had not returned to be screened at the recommended three-year interval. In the 2006 protocol, no active follow-up was included in the majority of centres, thus when the recommended interval is long (3 years) women may either forget the time of the next appointment or go to a private practice within a shorter interval. We do not know the underlying cause of such a failure to return but special action is needed to retain attenders. Further analysis at a longer interval is currently being done to further evaluate the spontaneous return to screening visits.

Overall, there was a very satisfactory evaluation of the introduction of the HPV test for its high validity in CIN2+ detection, and easiness to be implemented in laboratories of different complexity [10, 11]. Most likely our high profile in the detection of CIN2+ lesions included prevalent lesions together with incident ones. We have not yet been able to evaluate the detection rate in the second round, but it is expected that it will be lower as only incident cases will be expected if the interval is wide enough to avoid detection of recent new infections [20–22].

Further, the results of the proficiency testing assessment among laboratories for the HPV test showed that the HC2 assay has a high interlaboratory concordance [12].

Given these satisfactory results, HPV testing seems a clear candidate to substitute by large the use of cervical cytology as the primary screening tool. However, it is relevant to consider that the extension of HPV testing in the general population as primary screening should be done in a controlled manner for proper implementation. Strict monitoring of age, intervals between the test and referral rates should be established together with regular quality controls [19]. It is recommended that both professionals and users have the necessary training to successfully adapt the use of this molecular-based technology. It is not easy to explain the need to extend intervals to avoid an over diagnosis of infections and this requires full awareness of the scientific data and confidence with the implementation of new tests. It is also important to restrict the testing to women bellow age 29 to avoid detection of mainly acute HPV infections likely to regress in younger women. In 2014, several countries have incorporated the HPV test as a screening tool together or not with cytology [17, 18]. In Spain, more than seven scientific societies have expressed their approval for its introduction under an organised structure [6].

Another key element in the evaluation of this protocol was the availability of the information systems at the primary health level [23]. The fact that medical records in primary care are now extensible computerised and that these data are flushed periodically in a database linked to a unique individual identifier offers important opportunities for analysis. However, there are still geographic differences in the use of these systems, and there are difficulties in obtaining some results automatically from pathology and/or microbiology hospital records [23]. However, it should be noted that these limitations, which may explain some of the regional differences in coverage, are in the process of systematisation and improvement.

When exploring organisational aspects, It was found that teams were multidisciplinary with an important contribution of midwives in the identification of women susceptible to screening, sampling and reporting of cytology results if negative.

The introduction of the protocol has been complemented by training activities in which a free online course was included for professionals on cervical cancer prevention with a good record of acceptance.

## Conclusion

Based on the results of this evaluation [23], the urgency to monitor the loss of follow-up of women who had participated in screening of cervical cancer is recommended. The organisation of screening as a population activity in which women are actively called to the screening visit is strongly recommended to ensure equitable access for the maximum number of women at risk. The scientific evidence is overwhelming and such action is in accordance with the recommendations of the Spanish scientific societies [6]. It is also important to improve and expand the use of computerised medical records to manage the screening activities and to monitor the participation of women at risk. It is important to consider the transition to the use of HPV testing as a primary screening test for a better longitudinal sensitivity for the detection of cervical pathology. Any population-based screening activity should follow strict quality control assurance in all the steps and should allow for a period of transition, limited in time and strictly monitored.

## Figures and Tables

**Table 1.** Women registered at primary health care centres (HCC) in Catalonia, those attended for any medical reason and those with a record of a cervical cytology for the period 2008–2011.

Age	Registered women at HCC	Attended women	% of attended within group of age	Women with cytology	% of women with cytology
15–24	272,731	204,378	24.874.9	67,693	33.1
25–39	687,663	512,870	32.874.6	225,504	44.0
40–65	875,038	729,235	32.183.3	280,685	38.5
66–69	93,512	87,115	14.793.2	13,760	15.8
>69	363,621	340,932	2.393.8	8,226	2.4
25–65	1,562,701	1,242,105	32,479.5	506,189	40.8
TOTAL	2,292,564	1,874,530	26.081.8	595,868	31.8

**Table 2.** Accuracy of the HPV test, cytology, and the combination of both tests for the CIN2+ prediction at 3 years, in women over 39 years old with criteria of being underscreened at entry (analysis based on 767 observations).

	Sensitivity (95% CI)	Specificity (95% CI)	Positive predictive value (95% CI)	Negative predictive value (95% CI)
VPH	94.4 (74.2–99.0)	88.8 (86.3–90.8)	16.8 (10.8–25.3)	99.8 (99.2–100.0)
Cytology	44.4 (24.6–66.3)	96.3 (94.7–97.5)	22.9 (12.1–39.0)	98.6 (97.4–99.2)
VPH + Cytology	94.4 (74.2–99.0)	87.0 (84.4–89.3)	15.2 (9.7–23.0)	99.8 (99.1–100.0)

Adapted from Ibañez *et al*. 2014 [7].

## References

[1] Bosch FX (2012). Comprehensive control of human papillomavirus infections and related diseases. Vaccine.

[2] Kitchener HC, Castle PE, Cox JT (2006). Achievements and limitations of cervical cytology screening. Vaccine.

[3] Bruni L Human papillomavirus and related diseases in the world. Summary report.

[4] Puig-Tintoré LM, Torné A, Alonso I (2008). Current techniques in screening for cervical cancer in Spain: updated recommendations. Gynecol Oncol.

[5] Puig-Tintoré LM (2008). Coverage and factors associated with cervical cancer screening: results from the AFRODITA study: a population-based survey in Spain. J Low Genit Tract Dis.

[6] Torné A (2014). Guía de cribado del cáncer de cuello de útero en España, 2014. Progresos de Obstetricia y Ginecología.

[7] (2007). Departament de Salut Direcció general de planificació i avaluació: protocol de les activitats per al cribratge del càncer de coll uterí a l’atenció primària. http://www20.gencat.cat/docs/cancer/MERY/HPV/protocol.pdf.

[8] Rodríguez-Salés V (2014). Coverage of cervical cancer screening in Catalonia, Spain (2008–2011). Gac Sanit.

[9] Rodríguez-Salés V (2013). Coverage of cervical cancer screening in Catalonia for the period 2008–2011 among immigrants and Spanish-born women. Front Oncol.

[10] Ibáñez R (2014). Protecting the underscreened women in developed countries: the value of HPV test. BMC Cancer.

[11] Ibáñez R (2012). Prediction of cervical intraepithelial neoplasia grade 2+ (CIN2+) using HPV DNA testing after a diagnosis of atypical squamous cell of undetermined significance (ASC-US) in Catalonia, Spain. BMC Infect Dis.

[12] Ibáñez R (2014). Interlaboratory reproducibility and proficiency testing within the human papillomavirus cervical cancer screening program in Catalonia, Spain. J Clin Microbiol.

[13] Solomon D (2002). Terminology for reporting results of cervical citology. JAMA.

[14] Departament de Salut Generalitat de Catalunya Canal Cancer (2014). http://cancer.gencat.cat/es/professionals/deteccio_precoc/cancer_de_coll_d_uter/.

[15] De Sanjosé S (2013). Avaluació del protocol de les activitats de cribratge de càncer de coll uterí a l’Atenció Primària a Catalunya. Informe Tècnic Barcelona. https://www.hpvcentre.net/cribratge/index_2.php.

[16] Acera A (2014). Analysis of three strategies to increase screening coverage for cervical cancer in the general population of women aged 60 to 70 years: the CRICERVA study. BMC Womens Health.

[17] Health council of the Netherlands (2011).

[18] Ronco G (2012). Health technology assessment report: HPV DNA based primary screening for cervical cancer precursors. Epidemiol Prev.

[19] Arbyn M (2010). European guidelines for quality assurance in cervical cancer screening. Second edition–summary document. Ann Oncol.

[20] Arbyn M (2012). Evidence regarding human papillomavirus testing in secondary prevention of cervical cancer. Vaccine.

[21] Rijkaart DC (2012). Human papillomavirus testing for the detection of high-grade cervical intraepithelial neoplasia and cancer: final results of the POBASCAM randomised controlled trial. Lancet Oncol.

[22] Ronco G (2014). International HPV screening working group. Efficacy of HPV-based screening for prevention of invasive cervical cancer: follow-up of four European randomised controlled trials. Lancet.

[23] de Sanjose S (2015). El cribado del cáncer de cuello de útero en el Sistema Público de Salud de Cataluña. Evaluación y seguimiento durante el perıodo 2006-2012. Prog Obstet Ginecol.

